# Exploring the long noncoding RNAs-based biomarkers and pathogenesis of malignant transformation from dysplasia to oral squamous cell carcinoma by bioinformatics method

**DOI:** 10.1097/CEJ.0000000000000527

**Published:** 2016-07-23

**Authors:** Hongcheng Jia, Xuan Wang, Zheng Sun

**Affiliations:** Departments of a Stomatology, Beijing Ditan Hospital; b Stomatology, Beijing Tongren Hospital; c Oral Medicine, Beijing Stomatological Hospital, Capital Medical University, Beijing, China

**Keywords:** biomarker, dysplasia, differentially expressed genes, long noncoding RNA, oral squamous cell carcinoma, transformation

## Abstract

Long noncoding RNAs (lncRNAs) play an important role in many biological processes and carcinogenesis. We aimed to explore lncRNA-based pathogenesis, diagnostic biomarkers, and predictive factors of malignant transformation from dysplasia to oral squamous cell carcinoma (OSCC). Microarray data of GSE30784 consisting of 167 OSCC, 17 dysplasia, and 45 normal oral tissues were downloaded from the GEO database. The differentially expressed genes (DEGs) and lncRNAs between the three samples were identified using R, followed by lncRNA-mRNA coexpression and coregulation network analysis for the prediction of lncRNA target genes. Gene Ontology and Kyoto encydopedia of gene and genomes pathway analysis were performed to further characterize potential interactions. A total of 4462 DEGs and 76 differentially expressed lncRNAs were screened between the three groups, and 200 DEGs and only double homeobox A pseudogene 10 (*DUXAP10*) were screened among the three groups. A total of 1662 interactions of 46 lncRNAs and their coexpressed target genes were predicted, and 38 pairs of lncRNA-lncRNA coregulated 843 target genes. The coregulated target genes significantly enriched in antigen adaptive immune response, activation of phagocytosis receptor signaling, mast granule NF-κB inflammation, etc. Overall, lncRNAs were differentially expressed in OSCC and dysplasia. The target genes might play an important role in the carcinogenesis and development of OSCC. These results improve our understanding regarding the lncRNA-based pathogenesis and identify some potential targets for early diagnosis of malignant transformation from dysplasia to OSCC.

## Introduction

Oral squamous cell carcinoma (OSCC) is one of the commonest cancers in the world, and its incidence and mortality have been increasing significantly in recent years (Sasahira *et al.*, 2014). Despite important and several advances in medical and surgical treatment of OSCC, the five-year survival rate is less than 50%. This is due to the fact that most of the patients are in middle and advanced stages of cancer during diagnosis, resulting in poor prognosis (Brocklehurst *et al.*, 2010). OSCC usually develops from precancerous lesions such as oral leukoplakia, erythroplakia, lichen planus, etc., and histopathologically it follows a step-wise pattern of hyperplasia, dysplasia, carcinoma in situ, and invasive squamous cell carcinoma. The progression might continue for few years to decades, and such patients require long-term follow-up for the prevention of malignant transformation to invasive cancer. Epithelial dysplasia is a key watch point in the progression. Although some tumor markers, such as serum squamous cell carcinoma antigen, carbohydrate antigen 19-9, and carcinoembryonic antigen have been used in the early diagnosis and monitoring of OSCC, their lack of sensitivity and specificity limited their clinical value (Irimie *et al.*, 2017). Therefore, it is an urgent clinical problem to search for biomarkers that can help detect high-risk lesions as early as possible so that more invasive lesions can be prevented from occurring.

Long noncoding RNAs (lncRNAs) are a novel class of RNA molecules that range from 200 nt to >100 kb in length. According to transcriptional profiling, the lncRNA expression was significantly abnormal in human tumors. Gibb *et al.* (2011) first reported the expression profiles of lncRNAs in human normal oral mucosa and oral epithelial dysplasia using serial analysis of gene expression. Of the 325 lncRNAs, 60% were abnormally expressed in oral epithelial dysplasia, including some lncRNAs that are differentially expressed in other human tumors, such as HOTAIR, MALAT1, etc. Moreover, many researchers have detected the expression of lncRNAs in OSCC tissues and normal tissues by PCR, and revealed that some lncRNAs were differentially expressed in normal tissues, tumor tissues, and tissues with tumor metastasis, and might act as potential markers of OSCC or used as independent biomarkers for predicting the prognosis of patients with OSCC (Tang *et al.*, 2013; Fang *et al.*, 2014; Jia *et al.*, 2014). However, few studies reported the association of lncRNAs to malignant transformation from dysplasia to OSCC, but its molecular pathological mechanism is still unclear.

Hence, in this study, we downloaded the data of lncRNA expression profiles from GEO database, identified the differentially expressed lncRNAs and DEGs by bioinformatics, constructed the coexpression of lncRNA-mRNA and coregulation network of lncRNA-lncRNA, and predicted the target genes of differentially expressed lncRNAs. We further explored the molecular mechanism of the occurrence and development of OSCC by functional enrichment analysis of the target genes. These findings can help in better understanding of the molecular mechanisms in lncRNA perspective, providing a basis for the diagnostic biomarkers and predictive factors of malignant transformation from dysplasia to OSCC to justify the intervention timing and measures.

## Materials and methods

### Affymetrix microarray data

The data (GSE30784) were downloaded from the GEO database (http://www.ncbi.nlm.nih.gov/geo/). The gene chip data were completed and submitted to GEO by Chen *et al.* (2008) from the Fred Hutchinson Cancer Research Center, which included 167 cases of OSCC, 17 cases of atypical hyperplasia, and 45 cases of normal control. The platform is GPL570, namely, the Affymetrix U133 2 Plus GeneChip arrays.

### Data preprocessing

GEOquery package in R language was used to obtain the gene chip expression data whose accession number is GSE30784, and its acquisition address is https://ftp.ncbi.nlm.nih.gov/geo/series/GSE30nnn/GSE30784/matrix/GSE30784_series_matrix.txt.gz. The dataset contains 229 samples and expression information of 54 675 probes. At the same time, the annotation information of the probes, such as the associated gene symbols, Entrez Gene ID and gene name, and the clinical annotation information of the sample, such as age, gender, and disease status (cancer, dysplasia and control), were also obtained.

The Gencode version 26 database includes detailed gene annotation information based on the genomic version GRCh37 (hg19), annotating the lncRNA gene. The downloading address of the dataset was http://www.gencodegenes.org/releases/26lift37.html. So, we searched the gene symbols corresponding to the probe in the Gencode dataset and labeled whether the probe was an lncRNA probe or not.

### Screening of differentially expressed long noncoding RNAs and differentially expressed genes

The differential expression of lncRNAs and genes were analyzed using R’s limma package (http://www.bioconductor.org/packages/release/bioc/html/limma.html). The multiple testing *P* values were corrected using Benjamini-Hochberg algorithm (Benjamini and Hochberg, 1995). The differentially expressed lncRNAs and DEGs were selected according to the change multiples of >2 or <1/2 and the corrected *P* value <0.05.

### Prediction of target genes of differentially expressed long noncoding RNAs

Based on the expression values of all 229 samples, Pearson correlation coefficients of lncRNAs and mRNAs that were significantly differently expressed between the three groups were calculated separately. If the correlation coefficient was greater than 0.8 and the correlation test *P* value was <0.05, the lncRNA-mRNA pair was defined for coexpression, and the mRNA was considered to be a target gene for lncRNA. If two lncRNAs are coexpressed with the same mRNA, then the lncRNA-lncRNA pair was defined for coregulation.

### Functional analysis of target genes regulated by long noncoding RNAs

Functional enrichment analysis of coregulated genes was done by Maere *et al.* (2005). Functional enrichment analysis of coexpressed genes was done by Huang *et al.* (2009). Functional networks were constructed by Enrichment Map plug in the Cytoscape.

## Results

### Differentially expressed genes and differentially expressed long noncoding RNAs

**Fig. 1:**
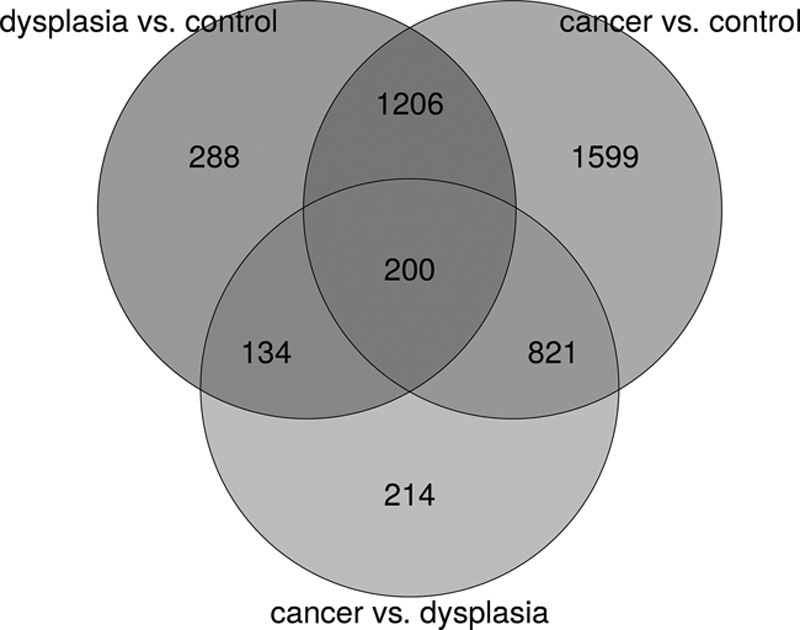
Venn picture of DEGs. The DEGs were filtered with foldchange >2 or <1/2, and the adjusted *P* value was <0.05. The number and overlap of DEGs between the three groups were compared. DEGs, differentially expressed genes.

**Fig. 2:**
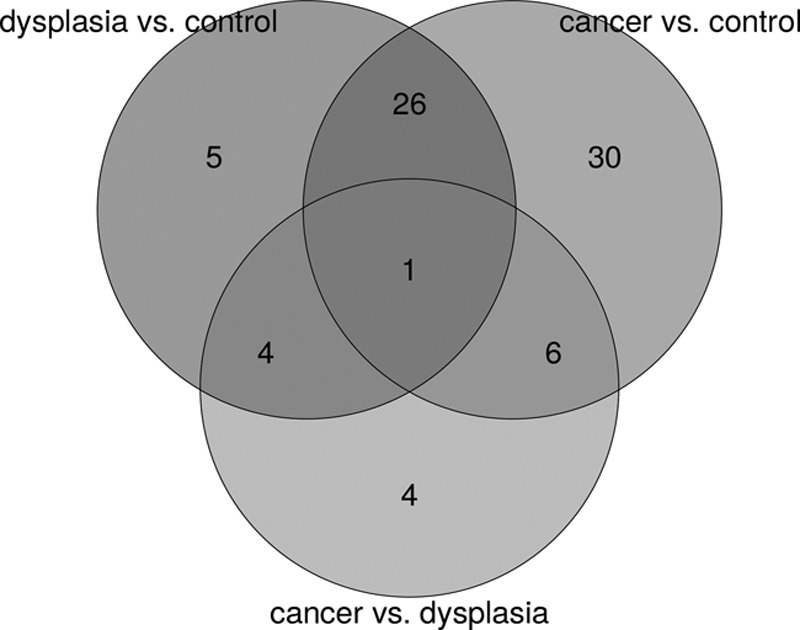
Venn picture of lncRNAs. The differentially expressed lncRNAs were filtered with foldchange >2 or <1/2, and the adjusted *P* value was <0.05. The number and overlap of lncRNAs between the three groups were compared. lncRNA, long noncoding RNAs.

**Fig. 3:**
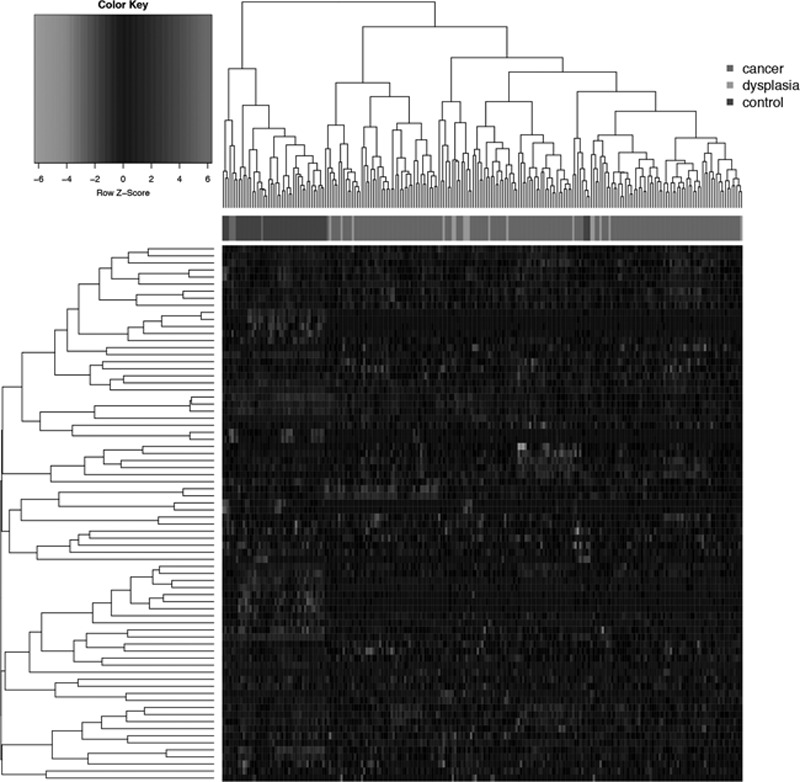
Heatmap of lncRNAs. Intersample distances are expressed using 1 – pearson correlation; distances between groups are calculated based on complete linkage, and clusters of samples and lncRNAs are hierarchically clustered. lncRNA, long noncoding RNAs.

**Fig. 4:**
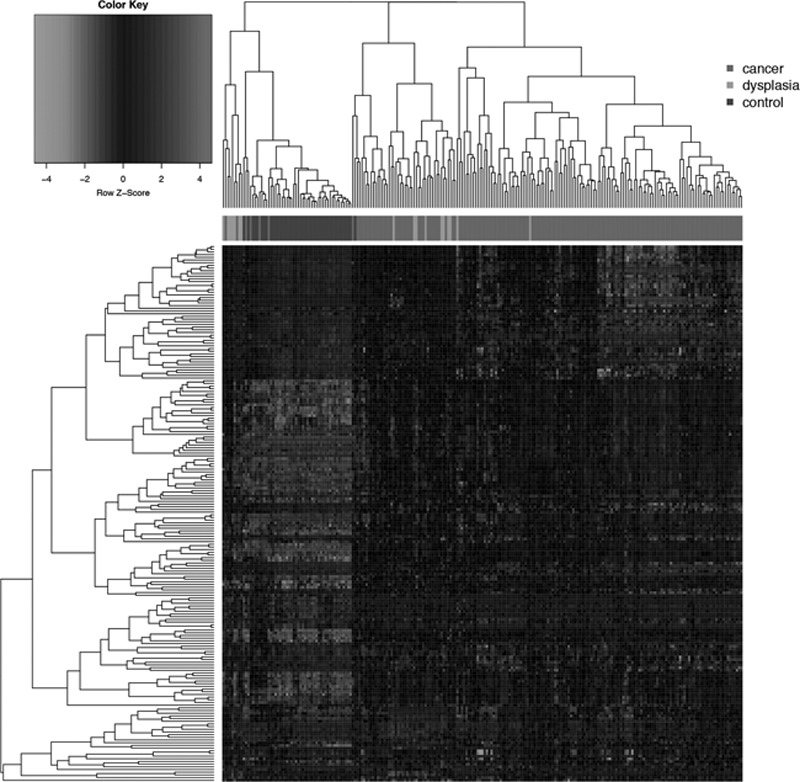
Heatmap of DEGs. Intersample distances are expressed using 1 – pearson correlation; distances between groups are calculated based on complete linkage, and clusters of samples and DEGs are hierarchically clustered. DEGs, differentially expressed genes.

A total of 3014 lncRNA probes and 51 661 gene probes were obtained through GENCODE annotation. Seventy-six differentially expressed lncRNAs and 4462 DEGs were screened according to the criteria between the three groups. There were 200 DEGs among OSCC, dysplasia, and normal epithelial tissues (Fig. 1), while there was only one differentially expressed lncRNA, that is, double homeobox A pseudogene 10 (*DUXAP10*) among the three groups was (Fig. 2). These differentially expressed lncRNAs and DEGs have a good clustering effect on OSCC, dysplasia, and normal epithelial tissues (Figs. 3 and 4).

### Target genes of long noncoding RNAs

**Fig. 5:**
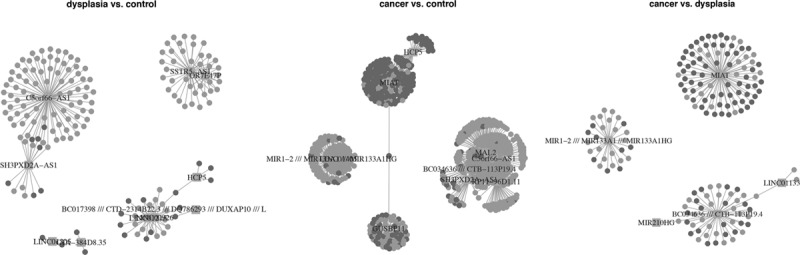
lncRNA-mRNA coexpression network. Orange square represents lncRNA, circle represents coexpressed gene, with red indicating upregulation and green indicating downregulation. lncRNA, long noncoding RNAs.

**Fig. 6:**
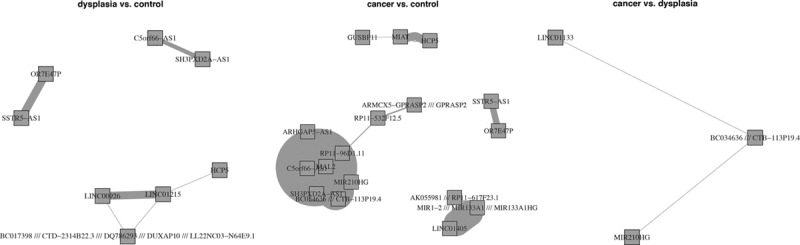
lncRNA-lncRNA coregulation network. The thickness of the line indicates the number of coregulated genes. lncRNA, long noncoding RNAs.

Between dysplasia and normal tissues, 15 lncRNAs were predicted to have coexpressed genes, and a total of 214 pairs of lncRNA-mRNA showed correlation (Table 1); six pairs of lncRNA-lncRNA coregulated 46 target genes (Table 2). Between OSCC and normal tissues, 26 lncRNAs were predicted to have coexpressed genes, and a total of 1287 pairs of lncRNA-mRNA showed correlation (Table 3); 30 pairs of lncRNA-lncRNA coregulated 795 target genes (Table 4). Between OSCC and dysplasia, five lncRNAs were predicted to have coexpressed genes, and a total of 61 pairs of lncRNA-mRNA showed correlation (Table 5); two pairs of lncRNA-lncRNA coregulated two target genes (Table 6). The coexpression and coregulation networks of differentially expressed lncRNAs and DEGs are shown in Figs. 5 and 6.

**Table 1:**
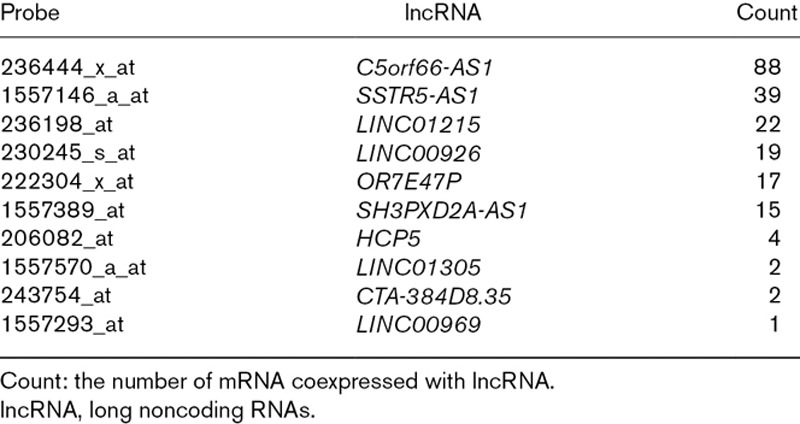
List of coexpressed long noncoding RNAs and the top 10 target genes in the group of dysplasia versus control

**Table 2:**
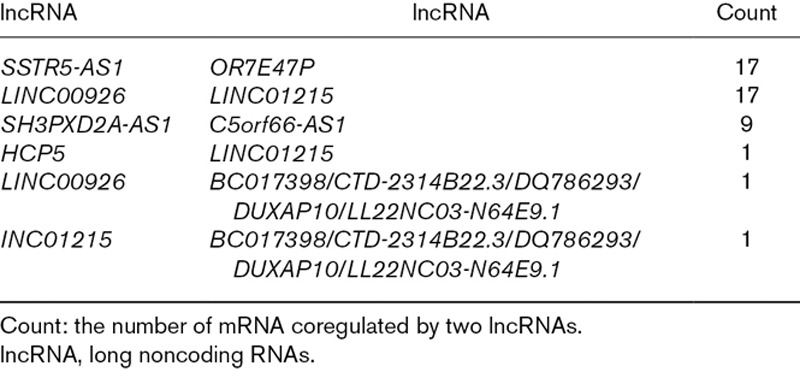
List of coregulated long noncoding RNAs and the target genes in the group of dysplasia versus control

**Table 3:**
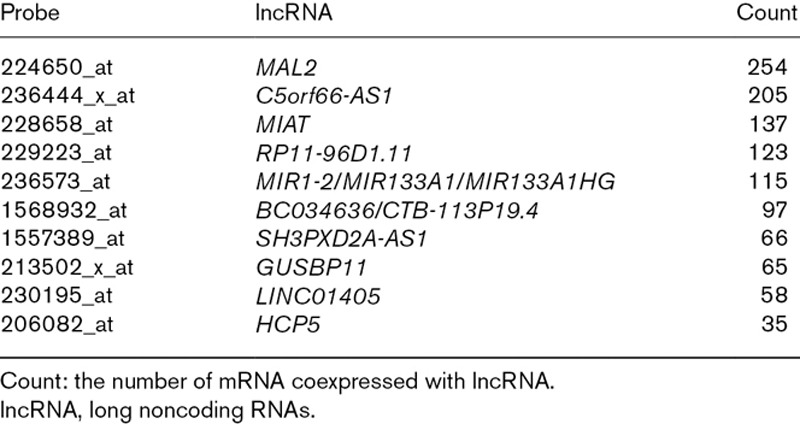
List of coexpressed long noncoding RNAs and the top 10 target genes in the group of cancer versus control

**Table 4:**
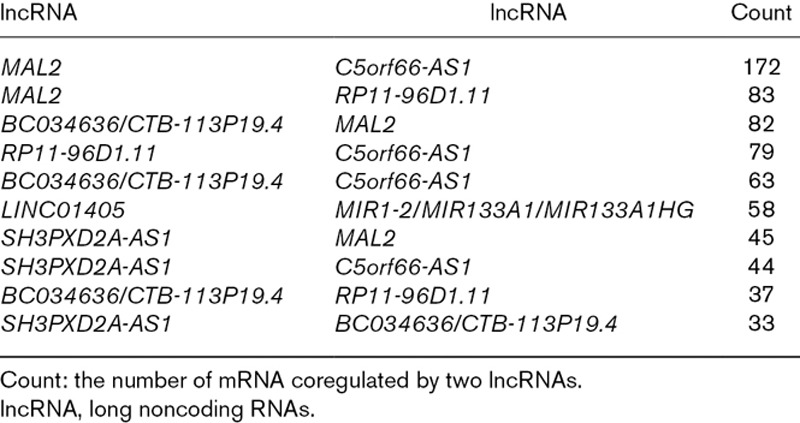
List of coregulated long noncoding RNAs and the top 10 target genes in the group of cancer versus control

**Table 5:**
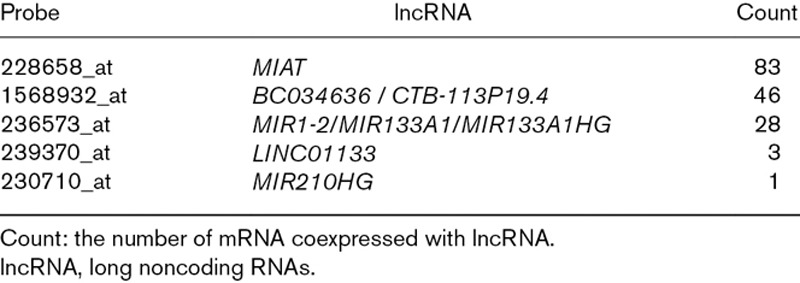
List of coexpressed long noncoding RNAs and the target genes in the group of cancer versus dysplasia

**Table 6:**
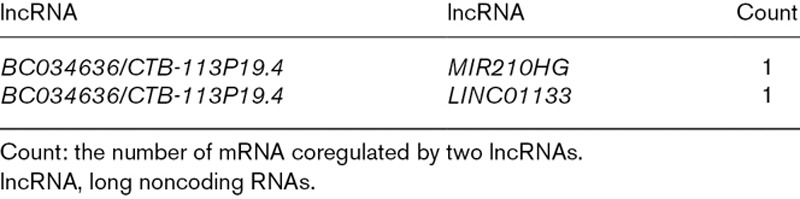
List of coregulated long noncoding RNAs and the target genes in the group of cancer versus dysplasia

### Gene Ontology function and Kyoto encydopedia of gene and genomes enrichment findings

**Fig. 7:**
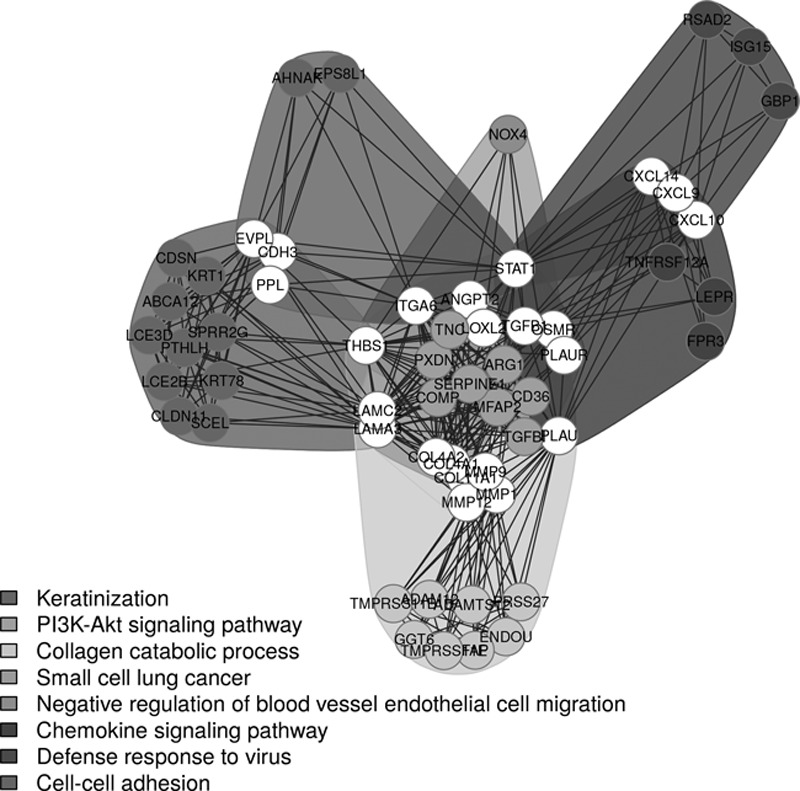
DEGs functional clustering network diagram. DEGs, differentially expressed genes.

**Fig. 8:**
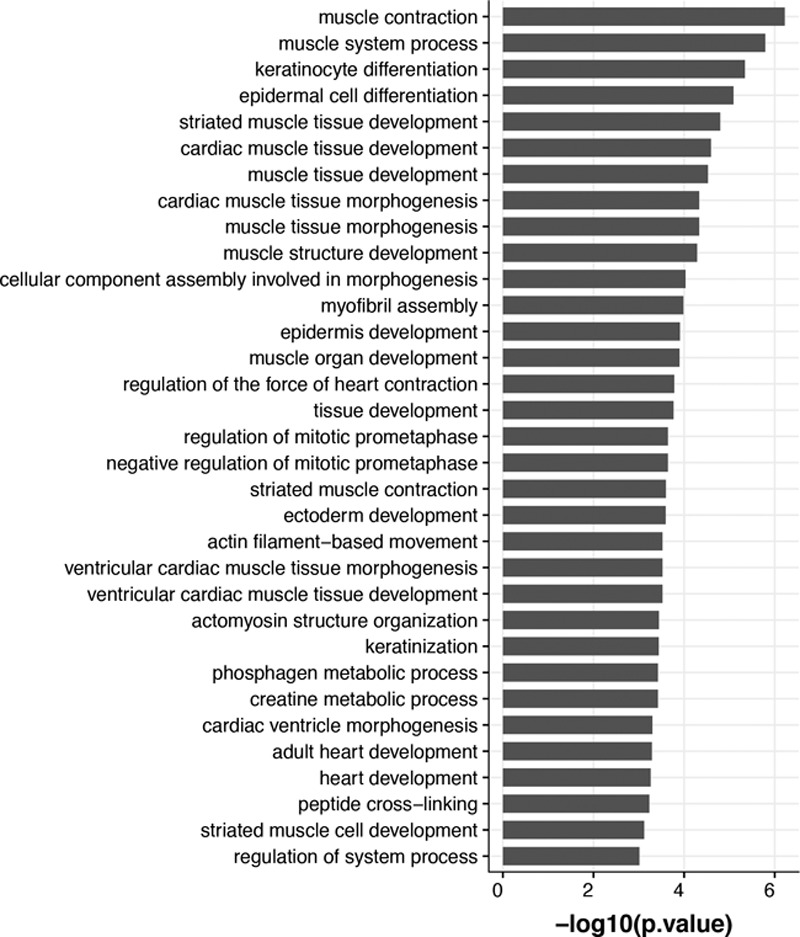
Coregulated gene function annotation network diagram.

**Fig. 9:**
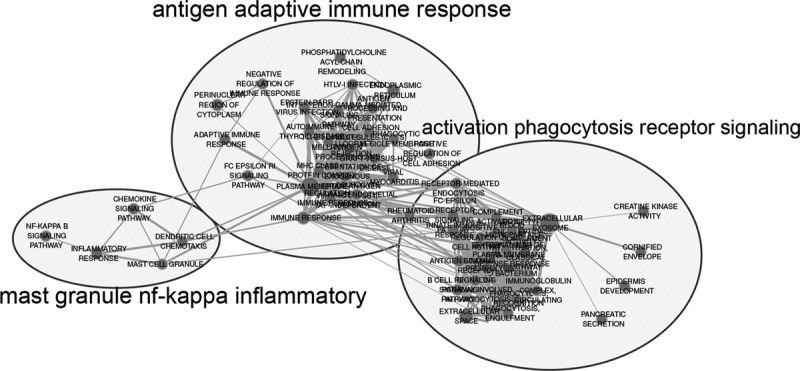
Functional enrichment analysis of DEGs coexpressed with differentially expressed lncRNAss. DEGs, differentially expressed genes.

The functions of 200 DEGs mainly include keratinization enrichment, phosphatidylinositol 3-kinase (PI3K)-Akt signaling pathway, collagen catabolic process, small cell lung cancer, negative regulation of blood vessel endothelial cell migration, and chemical signaling pathway, defense in response to virus, cell-cell adhesion, etc. (Fig. 7). The coregulated DEGs by differentially expressed lncRNAs mainly function in the enrichment of keratinocyte differentiation, epidermal cell differentiation, cellular component assembly involved in morphogenesis, tissue development, regulation of mitotic prometaphase, ectoderm development, keratinization, etc. (Fig. 8). The functions of DEGs coexpressed with differentially expressed lncRNAs are mainly enriched in antigen-adaptive immune response, activation of phagocytosis receptor signaling, and mast granule NF-κB inflammation (Fig. 9).

## Discussion

OSCC has a high degree of malignancy and poor prognosis, seriously affecting the quality of life of patients. Although majority of precancerous lesions do not transform into OSCC, it is imperative to identify high-risk lesions. The malignant transformation involves accumulation of genetic changes. Recognition of aberrant genes is beneficial for the early detection and prevention of transforming lesions. This study focused on lncRNA expression in different stages of progression and the crucial lncRNAs that could be used as biomarkers were filtered for accurate detection of high-risk lesions. With good histopathological guidance, a reliable and objective molecular marker that can help clinicians to take right clinical decisions regarding high-risk lesions without undergoing biopsies, and acceptable by patients is required.

In this study, microarray data of OSCC, dysplasia, and normal tissue samples were selected to identify the lncRNAs related to carcinogenesis of OSCC, construct a coexpression network of lncRNA-mRNA, and predict the target genes regulated by lncRNA based on coexpression results. Functional enrichment analysis of target genes was carried out to understand the synergistic effect of risky lncRNAs on malignant transformation of OSCC and their functional impact on the body. Seventy-six differentially expressed lncRNAs were screened based on the microarray data. The results of functional enrichment analysis of target genes showed that the synergistic effect of coregulatory target genes of risky lncRNAs was reflected in the tumor microenvironment changes such as immune and inflammatory responses, confirming that the corresponding risk indicator of lncRNAs screened by bioinformatics method and the prediction of target genes regulated by lncRNAs based on coexpression relationship were credible. These differentially expressed lncRNAs and DEGs were suspected to be involved in the carcinogenesis and progression of OSCC.

LncRNA is widely involved in many biological processes and is differentially expressed in tumors. Hence, many studies have focused on whether the lncRNA can be used as a potential tumor marker in clinical diagnosis (Hanahan and Weinberg, 2011; Fatima *et al.*, 2015) of OSCC (Tang *et al.*, 2013; Zhang *et al.*, 2015). Gibb *et al.* (2011) analyzed SAGE libraries from normal oral mucosa and abnormal hyperplasia samples, and revealed that *NEAT1* had the highest expression in SAGE expression profiles whether in normal mucosa (1305 TPM) or abnormal hyperplasia tissues (739 TPM). *NEAT1* is also highly expressed in the saliva of OSCC patients (Tang *et al.*, 2013). It plays an important role in the carcinogenesis of OSCC and has potential to act as a predictive biomarker for tailoring clinical decision in patients with high-risk lesions (Jia *et al.*, 2018). Similarly, we initially found that *DUXAP10* was differentially expressed in OSCC, dysplasia, and normal oral epithelial tissues, and was believed to be associated with the occurrence and progression of OSCC, typifying the high-risk lesions. Theoretically, the differential expression levels of *DUXAP10* in different stages of OSCC can help clinicians to monitor the progression of OSCC.

LncRNA *DUXAP10* is a pseudogene with a length of 2398 nt and positioned at 14q11.2. Wei *et al.* (2017) for the first time reported its biological function and high expression pattern in small cell lung cancer. *DUXAP10* promoted the biological progression of small cell lung cancer by binding with LSD1 and epigenetic silencing of *LATS2* and *RRAD* expression. *DUXAP10* can inhibit *LATS1* expression by interacting with *PRC2* and *LSD1*, and maintain the stability of β-catenin mRNA by binding with *HuR*, thus promoting the development of gastric cancer. The high expression of *DUXAP10* is associated with poor prognosis of gastric cancer (Xu *et al.*, 2018). *DUXAP10* can regulate the proliferation and metastasis of pancreatic cancer cells by interacting with RNA-binding proteins EZH2 and LSD1. Knock out of *DUXAP10* can inhibit proliferation, migration and invasion of pancreatic cancer cells and promote their apoptosis (Lian *et al.*, 2018). Also it can inhibit PI3K/Akt/mTOR signaling pathway, reduce the expression of Bcl-xL, cyclin D, and CDK4 in bladder cancer cells, and increase the expression of Bad, cleaved caspase-3, cleaved caspase-9 and p27 (Lv *et al.*, 2018). *DUXAP10* is upregulated in colon cancer, and is associated with tumor progression, tumor size and lymphatic metastasis. It promotes tumor progression through epigenetic silencing of *p21* and *PTEN* (Lian *et al.*, 2017). These findings suggested that *DUXAP10* can be a candidate marker for multiple tumor diagnosis and target of gene therapy. The specific mechanism and differential expression pattern underlying the malignant transformation from dysplasia OSCC requires further investigation to provide new ideas for the early diagnosis and prevention of OSCC.

In general, the diagnosis, treatment, and prevention of diseases are advanced by our understanding regarding the underlying molecular events that drive these diseases. To decipher the role of lncRNAs in the molecular pathogenesis of OSCC transformation, we performed further investigation on lncRNA-gene coexpression network. Among all the functions enriched by lncRNA target genes in OSCC, PI3K-Akt signaling pathway and mast cell NF-κB signaling pathway are mostly related to cancer research. Chronic inflammation, which acts as a promotive factor in tumor microenvironment, is closely related to tumorigenesis and development (Balkwill and Mantovani, 2001; Coussens and Werb, 2002). How inflammatory cells regulate inflammation in tumor microenvironment is not fully understood, but mast cells as important regulators and participants of inflammation have attracted much attention (Kinet, 2007; Metz *et al.*, 2007). Several previous studies (Elpek *et al.*, 2001; Dabiri *et al.*, 2004; Rojas *et al.*, 2005) have revealed the vital role of mast cells in tumors. Mast cells are aggregated in tumors, suggesting that they may play an important role in the inflammation of tumors. Inflammation occurs in the tumor microenvironment, which in turn accumulates a large number of cytokines, chemical mediators and enzymes, such as TNF-a, IL-6, VEGF, iNOS, Cox-2, and MMP-9, for mediating inflammation and driving its malignant transformation (Gasparini *et al.*, 2003; Balkwill *et al.*, 2005; Kulbe *et al.*, 2007; Lawrence, 2007; Lin and Karin, 2007). These small molecules can be produced by mast cells, and then can enter the tumor tissues. These cells can reconstruct the microenvironment by regulating inflammation and immune responses. NF-κB activity is enhanced, and then induced the expression of cyclin to promote the proliferation of cancer cells (Huang *et al.*, 2008). The classical signaling pathway is composed of PI3K signaling and downstream protein kinase B (Akt) regulates proliferation, differentiation, cell cycle, DNA repair, protein synthesis, glycometabolism, angiogenesis, and cell migration of cancer cells. Its abnormal activity not only leads to malignant transformation of cells but also leads to tumor cell migration, adhesion, angiogenesis, and extracellular matrix degradation (Saji *et al.*, 2005; Manning and Cantley, 2007; Hers *et al.*, 2011; Martelli *et al.*, 2012). These results provided quite a few suggestions regarding the possible biological functions of differentially expressed lncRNAs and DEGs. Using these mechanisms, the target genes with gain or loss of some special function were designed and goal of OSCC prevention can be achieved.

In conclusion, there are differential expressions of lncRNAs in OSCC and precancerous lesions. These differentially expressed lncRNAs and DEGs may participate in the occurrence and development of OSCC through PI3K-Akt signaling and mast cell NF-k functional pathways. Based on the understanding of these molecular events, it is possible for the early detection of high-risk lesions, monitoring the malignant transformation, and preventing the OSCC using lncRNAs associated with carcinogenesis. Although the analysis of high throughput datasets is a complicated process, it is considered as a potentially very rewarding approach, especially in lesions with low tissue volume/availability. Furthermore, these findings warrant further verification.

## Acknowledgements

We would like to thank all the participants and research staffs in Capital Medical University and Medical Data Processing Center Peking Union Medical College Hospital for their invaluable contributions to this work.

## Conflicts of interest

There are no conflicts of interest.
